# The Study of the Response of Fat Metabolism to Long-Term Energy Stress Based on Serum, Fatty Acid and Transcriptome Profiles in Yaks

**DOI:** 10.3390/ani10071150

**Published:** 2020-07-07

**Authors:** Lin Xiong, Jie Pei, Xiaoyun Wu, Qudratullah Kalwar, Chunnian Liang, Xian Guo, Min Chu, Pengjia Bao, Xixi Yao, Ping Yan

**Affiliations:** 1 Animal Science Department, Lanzhou Institute of Husbandry and Pharmaceutical Sciences, Chinese Academy of Agricultural Sciences, Lanzhou 730050, China; xionglin@caas.cn (L.X.); peijie@caas.cn (J.P.); wuxiaoyun@caas.cn (X.W.); liangchunnian@caas.cn (C.L.); guoxian@caas.cn (X.G.); chumin@caas.cn (M.C.); baopengjia@caas.cn (P.B.); yaoxixi@caas.cn (X.Y.); 2 Key Laboratory for Yak Genetics, Breeding, and Reproduction Engineering of Gansu Province, Lanzhou 730050, China; 3 Department of Animal Reproduction, Shaheed Benazir Bhutto University of Veterinary and Animal Sciences, Sakrand 67210, Pakistan; qudratullahkalwar@gmail.com

**Keywords:** yak, fat metabolism, long-term energy stress, adenosine 5′-monophosphate-activated protein kinase (AMPK)

## Abstract

**Simple Summary:**

The serum, fatty acid and transcriptome profiles in the subcutaneous fat of yaks were measured to explore the effect of long-term energy stress (ES) on fat metabolism during the cold season. The study indicated that under long-term ES during the cold season, the amount of fat in yaks was less, and fat mobilization was one of the main ways by which energy was obtained in yaks. Yaks regulated fat metabolism in subcutaneous fat primarily through adenosine 5′-monophosphate-activated protein kinase (AMPK) signaling. Glucose (GLU) intake, fat catabolism, fatty acid synthesis and fatty acid oxidation in the subcutaneous fat of yaks were all inhibited, which resulted in the fat mobilization of yaks slowing as much as possible under long-term ES. In addition, the energy expenditures in fat cells were inhibited by regulating phosphatidylinositol 3’ -kinase (PI3K)-serine/threonine-protein kinase (Akt) andmammalian target of rapamycin (mTOR) signaling, and the limited energy obtained from GLU and fat was consumed by muscle and organs as much as possible. These factors led to an energy balance in yaks under long-term ES. The fat stored in yaks can be expended for as long as possible, and yaks can survive for as long as necessary under long-term ES.

**Abstract:**

Long-term energy stress (ES) during the cold season is a serious problem for the breeding of yaks. In this paper, the response of fat metabolism in yaks to long-term ES during the cold season was studied. Gas chromatography (GC) analysis showed that the percentage of saturated fatty acids (SFAs) in the subcutaneous fat of the yaks in the ES group was 42.7%, which was less than the 56.6% in the CO group (*p* < 0.01) and the percentage of polyunsaturated unsaturated fatty acids (PUFAs) in the subcutaneous fat of the yaks in the ES group was 38.3%, which was more than the 26.0% in the CO group (*p* < 0.01). The serum analysis showed that fatty acid oxidation in yaks was increased under long-term ES. In the subcutaneous fat of yaks under long-term ES, the gene expression levels of glycerol-3-phosphate acyltransferase 4 (GPAT4), hormone-sensitive lipase (HSL), patatin-like phospholipase domain-containing protein 2 (PNPLA2), acyl-CoA dehydrogenase (ACAD), acyl-coenzyme A thioesterase 8 (ACOT8), facilitated glucose transporter (GLUT4), 3-oxoacyl-[acyl-carrier-protein] synthase (OXSM), oestradiol 17-beta-dehydrogenase 8 (HSD17B8) and malonate-Co-A ligase ACSF3 (ACSF3) were downregulated (*q* < 0.05), whereas the gene expression levels of aquaporin-7 (AQP7), long-chain-fatty-acid-CoA ligase (ACSL), elongation of very long chain fatty acids protein (ELOVL) and fatty acid desaturase 1 (FADS1) were upregulated (*q* < 0.05), indicating the inhibition of fat catabolism, fat anabolism, fatty acid oxidation, glucose (GLU) intake and SFA synthesis and the promotion of glycerinum (GLY) transportation and PUFA synthesis. Additional findings showed that the gene expression levels of leptin (LEP), adenosine 5′-monophosphate-activated protein kinase (AMPK) and phosphatidylinositol 3-kinase (PI3K) were upregulated (*q* < 0.05), whereas the gene expression levels of malonyl-CoA decarboxylase (MCD), sterol regulatory element-binding protein 1 (SREBF1), mammalian target of rapamycin (mTOR) and serine/threonine-protein kinase (AKT) were downregulated (*q* < 0.05), indicating that fat metabolism in the subcutaneous fat of yaks under ES was mainly regulated by AMPK signaling and mTOR and PI3K-AKT signaling were also involved. Energy consumption was inhibited in the subcutaneous fat itself. This study can provide a theoretical basis for the healthy breeding and genetic breeding of yaks.

## 1. Introduction

Yaks (*Bos grunniens*) are unique livestock species which are mostly found in the Tibetan Plateau [1], and there are currently approximately 20 million yaks in the world. Yaks play important roles in the daily lives of local residents, including supplying animal-derived food, transport, shelter and fuel [2,3]. As a classic grazing livestock, the growth of yaks is directly affected by the natural environment. Because of the high altitude (more than 4000 m) of the Tibetan Plateau, there is only a cold season and a warm season [4]. The growth of animals is affected by the balance between the supply of food and the cost of survival each day [5]. During the cold season, from October to the following April, the grass withers and even dies, and yaks only obtain limited energy from it. In addition, more energy is used to handle the low temperature, and yaks suffer from energy stress (ES) for a long time. During the long period of life and evolution, yaks have formed a strong adaptability to long-term ES during the cold season. When the energy from cured hay cannot meet the needs of yaks, some nutrient substances that were previously stored in yaks are decomposed to supply energy.

Fat is an important molecule for energy storage in animals [6]. The regulation of fat metabolism is a complex process that exists in a cycle of fat anabolism, fat catabolism and energy generation [7,8]. The relative balance between fat anabolism and catabolism in adipose tissues controls the release of free fatty acids from fat cells, which is critical to ensure energy metabolism in animals. Increased fat mobilization in adipose tissues is the major metabolic adaptation to a negative energy balance [9]. Fat metabolism plays an important role in the survival and reproduction of yaks during cold seasons. However, to the best of our knowledge, some aspects of the seasonal restructuring of fat, the biochemical processes and the molecular background of fat metabolism in yaks under long-term ES during the cold season have not been explored. Fat metabolism is regulated by the expression of relevant genes in adipose tissue. Transcriptomic studies have been conducted to analyze differentially expressed genes (DEGs) and to explore physiological mechanisms in animals. In recent years, high-throughput RNA sequencing (RNA-seq) has developed very quickly and offers several advantages over other transcriptome profiling methods, such as microarray analysis or real-time PCR. RNA-seq has been performed to explore the mechanism and potential candidate genes related to fat metabolism in animals, including cattle [10,11], cows [12], chickens [13,14,15], zebrafish [16] and pigs [17,18]. Studying fat metabolism in yaks under ES during cold seasons with RNA-seq can offer a unique opportunity to understand how yaks adapt to long-term ES during the cold season by regulating fat metabolism at the transcriptome level.

In this study, the fat content and fatty acid profiles in yaks under long-term ES were explored. A transcriptomic approach, combined with serum profiling, was used to compare the molecular content of the fat in yaks under normal physiological states and under long-term ES to evaluate how gene expression influenced fat metabolism. In addition, bioinformatic analyses, including functional enrichment of gene ontology (GO) terms and Kyoto Encyclopedia of Genes and Genomes (KEGG) pathway analysis, were performed to determine the pathways and processes that were enriched in the differential expression profile. Finally, quantitative reverse transcriptase polymerase chain reaction (RT-PCR) was performed to validate the differential expression of the selected genes identified by RNA-Seq. This study can establish a theoretical basis for the improved breeding and healthy maintenance of yaks during the cold season.

## 2. Materials and Methods

### 2.1. Animals

The animal study was approved by the Ethics Committee of the Lanzhou Institute of Husbandry and Pharmaceutical Sciences, Chinese Academy of Agricultural Sciences. The experimental location was a pasture in Haiyan County (100°25′ E, 36°55′ N) in Qinghai Province, China. Twelve male yaks (four years old) with similar body weights (250 ± 10 kg) were randomly divided into the energy stress (ES) group and the control (CO) group, with six yaks in each group. The yaks in the CO group were kept in grazing conditions from July to September, while the yaks in the ES group were kept in grazing conditions from July to the following April. During the experiment, all the yaks were given free-choice access to food and water.

The feces of the yaks were collected by pockets and weighed five times each month, and the grass was collected in the middle of each month. The in vitro dry matter digestions (DMDs) of grass were measured according to the method described by Tilly and Terry [19]. The dry matter intakes (DMIs) of yaks were measured by the acid-insoluble ash technique. The metabolic energy intakes (MEIs) of yaks were calculated according to the method described in Takahashi et al. [20]. The results showed that the DMDs during the warm season and cold season were 53.2 ± 3.63% and 50.3 ± 5.89%, respectively, and these values were similar; the DMI in the ES group was 5.91 ± 0.21 kg/d, which was less than the 7.53 ± 0.11 kg/d in the CO group (*p* < 0.01); the MEI in the ES group was 39.4 ± 2.44 MJ, which was less than the 57.9 ± 3.34 MJ in the CO group (*p* < 0.01). It was verified that the yaks were under long-term ES from October to the following April.

### 2.2. Slaughter Procedure and Sample Collection

Slaughtering yaks during the same season would not have allowed for obtaining the targeted groups with and without ES under natural pasture conditions. When harvesting the animals in September, they are in a normal, unstressed condition due to the good availability of pasture in the preceding months. For the April harvest, animals underwent a feed shortage during the previous cold season and thus were in ES. Twenty milliliters of blood were collected from the jugular vein of each yak under fasting conditions into a non-anticoagulant tube. The tubes were incubated to allow the blood to coagulate before centrifugation at 3000× *g* for 15 min at 4 °C using a KL05R refrigerated centrifuge (Kaida Inc., Changsha, China), and then the serum samples were obtained and stored at −20 °C in a BCD-507WDPT refrigerator (Haier Inc., Qingdao, China). The yaks were weighed and humanely euthanized at a commercial abattoir (Haiyan, China). The longissimus dorsi (12th–13th rib level), liver and subcutaneous fat samples were quickly collected. Parts of the subcutaneous fat samples were placed in enzyme-free cryopreservation tubes and stored in liquid nitrogen, and the other samples were placed into sealed pockets and frozen at −20 °C in a BCD-507WDPT refrigerator (Haier Inc., Qingdao, China).

### 2.3. Determination of Fat Content and Fatty Acid Profiles

The thickness of the subcutaneous fat on the backs (on both sides of the midline of the dorsum at the 5–6 thoracic vertebrae) and on the waists (on both sides of the midline at the cruciate region) of the yaks were measured using a vernier caliper (Hengliang Inc., Shanghai, China) within 10 min after slaughter. The body fat rates (BFRs) of the visceral fat, including the perirenal fat, omentum majus, mesentery fat and fat around the liver, were evaluated by the weighing method. The fat contents in the muscles and livers of the yaks were determined according to the procedures set forth by the Association of Official Analytical Chemists (AOAC) [21]. The fat was extracted in a Soxtec 2050 Soxhlet apparatus (FOSS Inc., Hillerød, Denmark) using petroleum ether and then weighed.

The fatty acid profiles were determined according to the method described in Song et al. [22]. The subcutaneous fat was placed into a centrifuge tube with a plug and decomposed into fatty acids by basic hydrolysis using 2 mL of a 0.1 M sodium hydroxide methanol solution in a water bath at 60 °C for 1 h. Then, 2 mL of a boron fluoride–methanol solution were added to derive the fatty acids. Finally, the fatty acid methyl esters were extracted with 2 mL n-hexane and the solution was passed into a vial through a PTFE Millipore filter. A gas chromatography system (7890A, Agilent Corp., Santa Clara, CA, USA) coupled with a flame ionization detector (FID) and an Agilent J&WCP-Sil88 FAME capillary column (100 m × 0.25 mm, 0.20 μm) was used to determine the extracts. The nitrogen constant linear flow rate was 0.5 mL/min, and the split ratio was 1:100. The initial column temperature was held at 100 °C for 5 min, then increased to 180 °C at 8 °C/min and held for 9 min. Then, the temperature was increased to 230 °C at 1 °C/min and held for 15 min. The injector and detector temperatures were 260 °C and 280 °C, respectively. The fatty acid methyl esters were qualitatively determined by their retention times and quantitatively by their peak areas outside standard methods. The contents of the fatty acids were calculated based on these results.

### 2.4. Determination of Serum Profiles

The levels of glucose (GLU), cholesterol (CH), triglyceride (TG), high-density lipoprotein cholesterol (HDL), low-density lipoprotein cholesterol (LDL), non-esterified fatty acid (NEFA) and albumin (ALB) in the serum of yaks were determined by the colorimetric method on a BS-420 automatic biochemical analyzer (Mindry Inc., Shenzhen, China). The levels of glucagon (GC), insulin (INS), oxaloacetic acid (OA), acetyl-CoA carboxylase (ACAC), β-hydroxybutyric acid (BHBA), hormone-sensitive lipase (HSL) and adipose triglyceride lipase (ATGL) in the serum of yaks were measured with the enzyme-linked immunosorbent assay (ELISA) method. Bovine ELISA kits (Beijing Sino-UK Institute of Biological Technology, Beijing, China) were used in the present study. The polyclonal primary antibodies were generated by inoculating rabbits with peptide antigens of dairy cattle, and peptide antigens were used as standards in the ELISA kits for the determination of INS, GC, ACAC, HSL and AGTL. It was proved that INS, ACAC, GC, HSL and AGTL of cattle share a high homology with the counterparts of yaks, and the sequences of the antigenic peptides that produce the antibodies are very similar between dairy cattle and yaks. The sensitivity and reliability of the ELISA kits for the determination of INS, GC, ACAC, HSL and AGTL in the serum of yaks were validated by a large number of experiments. The variation coefficients of the ELISA kits for the determination of GC, INS, OA, ACAC, BHBA, HSL and ATGL were 0.10%, 1.20%, 2.36%, 2.68%, 1.89%, 1.16% and 0.16%, respectively, suggesting that these kits were appropriate for monitoring the above proteins and metabolites in the serum of yaks.

### 2.5. RNA Extraction, Read Mapping and Expression Annotation

Three subcutaneous fat samples stored in liquid nitrogen were randomly selected from each group for the following experiments. The frozen samples were ground into powder by a mortar and pestle in liquid nitrogen. The total RNA was extracted from all the samples using the RNeasy Lipid Tissue Mini Kit (Qiagen, Valencia, CA, USA). The purity and concentration of the extracted RNA were determined by UV absorbance at 230, 260, and 280 nm using a NanoDrop 2000 spectrophotometer (Thermo Fisher Scientific, Santa Clara, CA, USA). The RNA quality was evaluated by measuring the RNA integrity number (RIN) with an Agilent 2100 Bioanalyzer (Agilent Corp., Santa Clara, CA, USA), and the RIN values for all the samples ranged from 8.0 to 8.5.

The mRNA sequencing libraries were constructed using the TruSeq Stranded mRNA LTSample Prep Kit (Illumina, San Diego, CA, USA). These libraries were sequenced on the HiSeq X Ten Illumina sequencing platform (Illumina, San Diego, CA, USA), and 125-bp paired-end reads were generated. The raw data were processed using Trimmomatic to remove the adaptor sequences and low-quality reads. The clean reads were mapped to the reference genome using hisat2. In addition, the Q20, Q30 and GC contents of the clean reads were calculated to assess the quality of the clean reads.

The numbers of unique match reads in the RNA-seq analysis were normalized against fragments per kilobase per million (FPKM) to compute the gene expression levels. The FPKM value of genes was quantified using cufflinks, and the read counts of genes were obtained by htseq-count. The DEGs were identified using the DESeq R package (2012) functions “estimateSizeFactors” and “nbinom test”. To further investigate the biological processes associated with the DEGs, functional enrichment analysis, including GO and KEGG, were carried out to identify which DEGs were significantly enriched in GO terms and metabolic pathways at a *p*-value ≤ 0.05 compared with the whole transcriptome background. The GO enrichment and KEGG pathway enrichment analyses of the DEGs were performed using R, based on the hypergeometric distribution.

### 2.6. Validation of Differentially Expressed Genes

To confirm the reproducibility of the gene expression data obtained by mRNA-Seq, the relative expression levels of selected DEGs were verified by qPCR. The *SERPINE1*, *LIPE* and *CEBPA* genes are discussed in this paper and play an important part in the response of fat metabolism in yaks to long-term energy stress; the *ACSF2*, *FAU*, *BCKDHA*, *GSTM3* and *TMEM165* genes are also connected with fat metabolism. Therefore, the above eight genes were selected for validation in this study. Previous research indicated that the expression levels of the *β-actin* gene in different tissues of yaks are partially variable. However, a good linear relationship between the expression level of *β-actin* and the amount of total RNA in the specific tissues, especially adipose tissue, was obtained in the confirmatory experiments. Therefore, the *β-actin* gene was selected as a reference gene for qPCR in the subcutaneous fat of yaks. The gene names, gene sequences and primer sequences are listed in Table 1. The reverse transcription (RT) reaction volume was 10 μL, which contained 0.5 μg total RNA, 0.5 μL gDNA Remover, 5 μL 5× TransScript All-in-one SuperMix, 0.5 μL (10 μM) of each primer and nuclease-free water. The reactions were performed in a GeneAmp^®^ PCR System 9700 thermal cycler (Applied Biosystems, Foster City, CA, USA) for 15 min at 42 °C and 5 s at 85 °C. The primers used for qPCR were designed by Oebiothch Co., Ltd. (Shanghai, China) and synthesized by TSINGKE Co., Ltd. (Beijing, China). The 10 μL RT reaction mix was then diluted 10 times in nuclease-free water and stored at −20 °C. Real-time PCR was performed using a LightCycler^®^ 480 Real-time PCR Instrument (Roche, Switzerland) with a 10 μL PCR mixture that included 0.2 μL cDNA, 5 μL 2 × PerfectStart^TM^ Green qPCR SuperMix, 0.2 μL (10 μM) of each primer and 3.6 μL nuclease-free water. The reactions were incubated in a 384-well optical plate (Roche, Basel, Swiss) at 94 °C for 30 s, followed by 45 cycles of 94 °C for 5 s and 60 °C for 30 s. Each sample was analyzed in triplicate. The relative mRNA expression levels for each target gene were calculated using the 2^−^^ΔΔ*C*T^ method and normalized against the internal marker gene *β-actin*.

## 3. Results

### 3.1. Fat Content and Fatty Acid Profiles

The subcutaneous fat of the yaks under long-term ES was thinner (1.49 mm on the back and 2.16 mm on the waist) than in the yaks in control conditions (8.17 mm on the back and 12.2 mm on the waist) (*p* < 0.05). The total BFR of the visceral fat of the yaks in the ES group was 0.69%, which was lower than the 4.17% in the control group (*p* < 0.01), indicating that the long-term ES had a substantial effect on the BFR of the visceral fat. The fat content in the liver of the yaks in the ES group was 1.65%, which was lower than the 2.21% in the CO group (*p* < 0.05). The intramuscular fat content of the yaks in the ES group was 1.01%, which was lower than the 1.84% in the CO group (*p* < 0.05).

A total of 32 fatty acids were simultaneously detected in the subcutaneous fat of the yaks in the ES and CO groups, as shown in Table 2. These fatty acids included 17 saturated fatty acids (SFAs), seven monounsaturated fatty acids (MUFAs) and eight polyunsaturated fatty acids (PUFAs). The fatty acid composition in the subcutaneous fat of the yaks under ES was different from the CO group. The percentage of SFAs in the subcutaneous fat of the yaks in the ES group was 42.7%, which was less than the 56.6% in the CO group (*p* < 0.01), and the percentage of unsaturated fatty acids (UFAs) in the subcutaneous fat of the yaks in the ES group was 57.3%. Moreover, the percentage of PUFAs in the subcutaneous fat of the yaks in the ES group was 38.3%, which was more than the 26.0% in the CO group (*p* < 0.01), while there was no difference in the percentage of MUFAs between the two groups. The percentage of the SFAs/UFAs in the subcutaneous fat of the yaks in the ES group was 0.75%, which was less than the 1.30% in the CO group (*p* < 0.01), and the PUFA/SFA percentage in the subcutaneous fat of the yaks in the ES group was 0.90%, which was greater than the 0.46% in the CO group (*p* < 0.01).

### 3.2. Serum Profiles

The results of the serum profiles are shown in Table 3. The levels of GLU, INS, NEFA, TG, LDL and ALB in the serum of the yaks under ES were lower than those in the serum of the yaks under control conditions (*p* < 0.05 or *p* < 0.01). The levels of GC, OA, BHBA, HSL and ATG in the ES group were higher than those in the CO group (*p* < 0.05 or *p* < 0.01). There were no differences in HDLD, CH and ACAC between the ES and CO groups.

### 3.3. Identification of Differentially Expressed Genes

**Figure 1:**
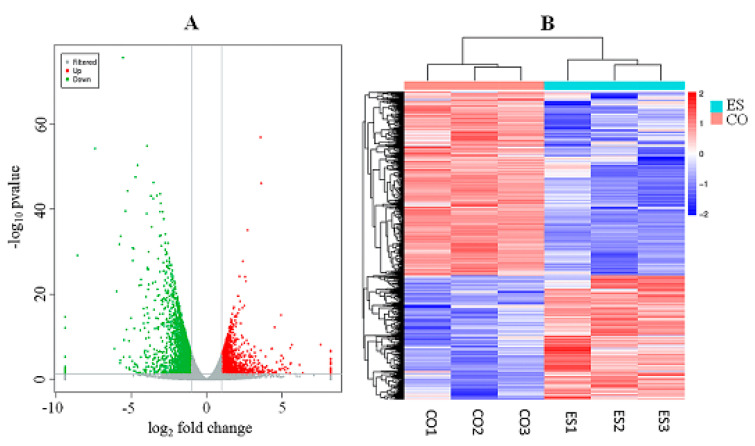
(**A**) Volcano plot of the total expression of genes in the subcutaneous fat of the yaks in the ES and CO groups. (**B**) Hierarchical clustering analysis of the transcriptome profiles in the subcutaneous fat of the yaks in the ES and CO groups.

In this study, genes with FDR < 0.05, *p* < 0.05 and |log2 (FC)| > 1 were considered as DEGs. The results indicated that 3480 DEGs were observed between the two groups. Of these DEGs, 1410 genes were upregulated, while 2070 genes were downregulated. The distribution of the DEGs is shown in a volcano plot in Figure 1A. In addition, a systematic cluster analysis of DEGs was performed to gain further insight into the similarities at the transcriptome level within the subcutaneous fat of yaks under long-term ES, as shown in Figure 1B. The individual samples belonging to the same group were clustered together. Furthermore, the patterns based on the relative expression levels of DEGs exhibited obvious differences between the ES and CO groups. These results suggested that the samples used in the present study were appropriately selected and that the grouping of these samples was reasonable.

### 3.4. GO and KEGG Analysis of DEGs

The DEGs were categorized into three major functional types based on GO analysis: biological process, molecular function and cellular component. A total of 1248 terms (*p* < 0.05 and the number of DEGs > 5) were significantly enriched in the three categories. The top 10 GO subgroups with the lowest *p*-values in the biological process were translation (GO:0006412), mitochondrial respiratory chain complex I assembly (GO:0032981), signal recognition particle-dependent co-translational protein targeting to membrane (GO:0006614), cytoplasmic translation (GO:0002181), translational initiation (GO:0006413), extracellular matrix organization (GO:0030198), collagen fibril organization (GO:0030199), mitochondrial electron transport (GO:0006120), NADH to ubiquinone (GO:0006120), anterograde axonal protein transport (GO:0099641) and UDP-N-acetylglucosamine biosynthetic process (GO:0006048). Based on KEGG pathway enrichment analysis, 89 KEGG pathways were significantly enriched (*p* < 0.05), and 17 KEGG pathways were related to fat metabolism, as shown in Table 4.

### 3.5. qPCR Validation

**Figure 2:**
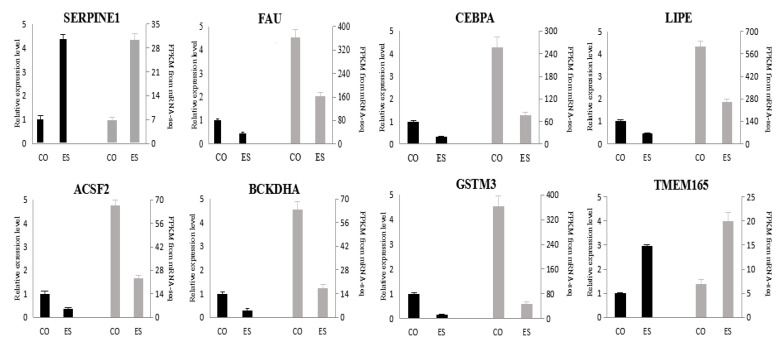
Validation of the *SERPINE1*, *FAU*, *CEBPA*, *LIPE*, *ACSF2*, *BCKDHA*, *GSTM3* and *TMEM165* genes by qPCR analysis.

Eight genes, namely *SERPINE1*, *FAU*, *CEBPA*, *LIPE*, *ACSF2*, *BCKDHA*, *GSTM3* and *TMEM165*, were selected for analysis. The results are shown in Figure 2, and all the selected DEGs showed concordant expression patterns between the mRNA-seq and qRT-PCR analyses.

## 4. Discussion

### 4.1. The Physiological Status of Yaks under Long-Term ES

The fat in yaks was less after long-term ES (*p* < 0.05), which indicated that fat mobilization was one of the main ways by which yaks obtained energy under ES. ALB is a macromolecule with important physiological functions in animals [23], and malnutrition can lead to lower levels of ALB. The ALB level in the serum of the yaks in the ES group was 26.5 g/L, which was lower than the normal level of 31.2 g/L in the CO group (*p* < 0.01). This result indicated that yaks under long-term ES were undernourished. The GLU level in the serum of the yaks in the ES group was 3.84 mmol/L, which was lower than the 4.95 mmol/L in the CO group (*p* < 0.01), indicating that the energy generated by GLU from grass cannot maintain the normal physiological function of yaks under long-term ES. The level of GC, which increases GLU, was 89.2 ng/mL in the serum of the yaks in the ES group, which was higher than the 50.3 ng/mL in the CO group (*p* < 0.01). These results indicated that the yaks under long-term ES need higher levels of GLU. INS is the only hormone that can reduce GLU in vivo, and the INS level in the serum of the yaks in the ES group was 9.53 ng/mL, which was lower than the 12.0 ng/mL in the CO group (*p* < 0.01). The levels of TG and NEFA in the serum of the yaks in the ES group were 0.19 mmol/L and 174 nmol/L, which were lower than the 0.25 mmol/L (*p* < 0.01) and 180 nmol/L (*p* < 0.05) in the CO group, respectively; these results indicated that the yaks in the ES group obtained less fat from inferior grass during the cold season. HSL and ATGL are the rate-limiting enzymes of fat catabolism. The levels of these enzymes in the serum of the yaks in the ES group were 12.2 and 66.9 ng/mL, which were higher than the 9.75 (*p* < 0.05) and 52.6 ng/mL in the CO group (*p* < 0.01), respectively; these results indicated that fat catabolism in the muscles and organs of yaks was more under ES. Both OA and BHBA are metabolites of fatty acid oxidation [24]. The levels of these metabolites in the serum of the yaks in the ES group were 13.62 and 4.26 mg/100 mL, which were higher than the 11.2 and 2.15 mg/100 mL in the CO group (*p* < 0.01), respectively; these results indicated that fatty acid oxidation in the muscles and organs of yaks was more to compensate for the lack of energy under ES.

### 4.2. The Effect of Long-Term ES on Fat Metabolism in Yaks

**Figure 3:**
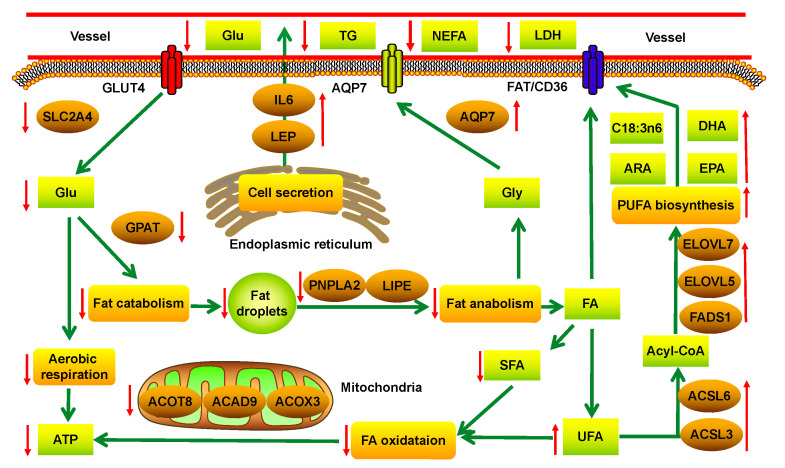
The fat metabolism in the subcutaneous fat of yaks under long-term energy stress (ES). ↑: upregulation of gene expression or increased concentration of metabolite or enhanced metabolic pathway; ↓: downregulation of gene expression or decreased concentration of metabolite or diminished metabolic pathway.

Fat metabolism in the subcutaneous fat of the yaks under long-term ES is shown in Figure 3. Glycerol-3-phosphateacyltransferase (GPAT) is the key catalyst in the production of fat [25]. The expression of the GPAT4 gene was downregulated in the subcutaneous fat of the yaks in the ES group (*q* < 0.01), and it was inferred that fat anabolism in the subcutaneous fat of the yaks under ES was inhibited. Hormone-sensitive lipase (HSL) and adipose triglyceride lipase (ATGL) are the rate-limited enzymes in fat catabolism [26,27]. The expression levels of the LIPE and PNPLA2 genes that encode these enzymes were also downregulated in the subcutaneous fat of the yaks in the ES group (*q* < 0.01). Therefore, fat catabolism in the subcutaneous fat of the yaks under ES was also inhibited. The GC analysis showed that the percentage of SFAs in the subcutaneous fat of yaks was 42.69% in the ES group, which was less than in CO group (*p* < 0.01), and it can be inferred that the fat derived from SFAs in the subcutaneous fat of yaks was preferentially used under ES during the cold season. AQP7 can transfer glycerinum (GLY) from fat catabolism to the cell membrane [28]. The expression of the AQP7 gene was upregulated in the subcutaneous fat of the yaks under ES (*q* < 0.05), and more GLY-derived fat catabolism in the subcutaneous fat of the yaks under ES can be transferred into the blood and used by muscles and organs via gluconeogenesis. Fatty acid oxidation includes four steps. Dehydrogenation is catalyzed by the acyl-CoA dehydrogenase (ACAD) family member [29,30]. The expression of the ACAD8 and ACAD9 genes was downregulated in the subcutaneous fat of the yaks under ES (*q* < 0.01); thiolysis is catalyzed by the acyl-CoA thioesterase (ACOT) family member [31]. The expression of the ACOT8 gene was also downregulated in the subcutaneous fat of the yaks under ES (*q* < 0.01). It was inferred that fatty acid oxidation was also inhibited in the subcutaneous fat of the yaks under ES.

The level of energy influences not only the amount of deposited fat but also its fatty acid profile [32]. The effect of animal energy on fatty acid composition has been demonstrated in several studies [33,34]. The enzymes 3-Oxoacyl-[acyl-carrier-protein] synthase (OXSM), oestradiol 17-beta-dehydrogenase 8 (HSD17B8) and malonate-CoA ligase ACSF3 (ACSF3) are involved in the positive regulation of the synthesis of SFAs, such as decanoic acid, dodecanoic acid, tetradecanoic acid, hexadecenoic acid and octadecanoic acid [35,36]. The expression levels of the *OXSM*, *HSD17B8* and *ACSF3* genes were downregulated in the subcutaneous fat of the yaks under ES (*q* < 0.05). Acetyl-CoA acyltransferase 2 (ACAA2) is the key enzyme in the process of translating acetyl-CoA to fatty acids (4 < n < 16) [37], and the expression of the *ACAA2* gene was downregulated (*q* < 0.01) in the subcutaneous fat of the yaks in the ES group. It was inferred that SFA synthesis was inhibited in yaks under ES during the cold season. Animals are incapable of synthesizing PUFAs endogenously and must obtain them from their diet. In addition to supplying energy to muscles and organs by fat catabolism, PUFAs play important roles in metabolism and maintaining normal cellular features. The percentages of n-3 and n-6 family PUFAs in the subcutaneous fat of yaks under ES, such as C20:4n6 (ARA) and C18:3n6, were higher than in CO group (*q* < 0.01). Long-chain acyl-CoA synthetases (ACSLs) are the rate-limited enzymes of fatty acid activation. The expression levels of the *ACSL3* (*q* < 0.01) and *ACSL6* genes (*q* < 0.05) [38] were upregulated in the subcutaneous fat of the yaks under ES, and it was inferred that PUFA synthesis was promoted in the subcutaneous fat of the yaks under ES. The elongases of very long chain fatty acids (ELOVLs) are key rate-limiting enzymes in long-chain fatty acid biosynthesis [39,40], and the expression of the *ELOVL7* (*q* < 0.01) and *ELOVL5* genes (*q* < 0.05) was upregulated in the subcutaneous fat of the yaks under ES. Fatty acid desaturase 1 (FADS1) [41] is the master regulator of n-3 and n-6 family PUFA synthesis, and the expression of the *FADS1* gene was also upregulated in the subcutaneous fat of the yaks under ES (*q* < 0.05). The synthesis of n-3 and n-6 family PUFAs was promoted, and the percentages of C22:6n3 (DHA), C20:5n3 (EPA), C20:3n3 and C20:4n6 (ARA) in the subcutaneous fat of yaks were all increased (*p* < 0.01). The n-6 and n-3 family PUFAs are the main constituents of cell membranes [42] and are also used to synthesize many bioactive substances, such as prostaglandin. ES can lead to the loss of n-3 and n-6 family PUFAs in the cellular membrane of yaks during the cold season, and more UFAs were synthesized to repair cell damage in yaks.

Fat metabolism in animals is regulated by certain adipocytokines. Lipoprotein lipase (LPL) can catalyze the decomposition of chylomicron-TG and VLDL-TG, and the expression of the LPL gene was upregulated in the subcutaneous fat of the yaks under ES (*q* < 0.05). When the yaks were under ES, more LPL was secreted by fat cells and entered the blood, and the utilization of fat from grass in the blood was increased. When animals are hungry, fat cells can secrete leptin (LEP), which decreases fat catabolism and conserves energy [43], and the expression of the *LEP* gene (*q* < 0.05) was upregulated in the subcutaneous fat of the yaks under ES. Angiopoietin-related protein 4 (ANGPTL4) can participate in the regulation of energy balance in animals [44,45], especially fat metabolism, and the expression of the *ANGPTL4* gene was downregulated in the subcutaneous fat of the yaks under ES (*q* < 0.01). A high level of interleukin-6 (IL-6) can slow the rate of the reaction in which triglycerides are converted into fatty acids. The expression of the *IL-6* gene was upregulated in the subcutaneous fat of the yaks under ES (*q* < 0.05). These results indicated that yaks try their best to decrease fat catabolism in subcutaneous fat and increase fat intake from grass by regulating the LPL, LEP, ANGPTL4 and IL-6 genes under long-term ES.

### 4.3. The Mechanism of the Response of Fat Metabolism to Long-Term ES

Adenosine monophosphate-activated protein kinase (AMPK) has a central role in the regulation of energy metabolism [46,47]. The serine/threonine-protein phosphatase 2A 65 kDa regulatory subunit A alpha isoform (PP2A) can inhibit the activation of AMPK by dephosphorylation, and the expression of its gene, PPP2R1A, was downregulated in the subcutaneous fat of the yaks under ES (*q* < 0.01). LEP can activate AMPK, and the expression of the LEP gene was upregulated in the AT of the yaks under ES (*q* < 0.05). The facilitated glucose transporter (GLUT4) can transit GLU via the cytomembrane, and the expression of its genes, *SLC2A3* (*q* < 0.05) and *SLC2A4* (*q* < 0.01) was downregulated in the subcutaneous fat of the yaks under ES, which indicated that fat cells can obtain less GLU from blood. On the other hand, fatty acid oxidation was inhibited in the subcutaneous fat of the yaks under ES. The main energy sources that can generate ATP were decreased in the subcutaneous fat of the yaks under ES. The expression of all 63 DEGs in the KEGG pathway of oxidative phosphorylation (bom00190), such as the NADH dehydrogenase, ATPase and oxidase genes, was downregulated in the subcutaneous fat of the yaks under ES (*q* < 0.05). It was inferred that ATP synthesis was inhibited and ADP/ATP increased in the subcutaneous fat of the yaks under ES. Calcium-binding protein 39-like (CAB39L) can activate AMPK by phosphorylation [48], and the expression of the *CAB39L* gene was upregulated in the subcutaneous fat of the yaks under ES (*q* < 0.01). All of these factors activated AMPK, and the expression of its gene, PRKAA1, was upregulated in the subcutaneous fat of the yaks under ES (*q* < 0.01).

**Figure 4:**
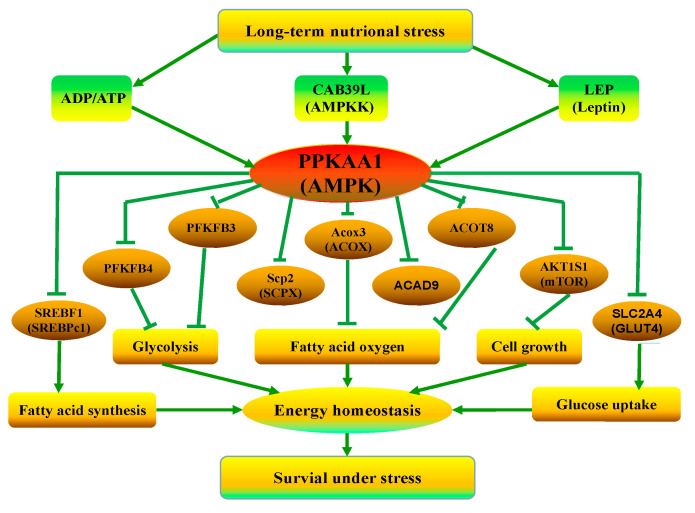
The energy balance in yaks under ES by regulating fat metabolism. →: promote or result in; ┴: repress gene expression, diminish metabolic pathway or inhibit physiological process.

To achieve energy balance under long-term ES, yaks regulated fat metabolism by the expression of the downstream genes involved in AMPK signaling [49], as shown in Figure 4. The expression of the *SLC2A3* (*q* < 0.05) and *SLC2A4* (*q* < 0.01) genes was downregulated in the subcutaneous fat of the yaks under ES, which indicated that less GLU from grass was translated into subcutaneous fat, and the muscles and organs could obtain limited GLU [50]. The expression of the LIPE gene was downregulated in the subcutaneous fat of the yaks under ES (*q* < 0.01), and fat catabolism in the subcutaneous fat of the yaks was inhibited. Sterol regulatory element binding transcription factor (SREBF1) is an important nuclear-encoded transcription factor that is related to fatty acid synthesis [51], and the expression of the *SREBF1* gene was downregulated in the subcutaneous fat of the yaks under ES (*q* < 0.01), indicating that fatty acid synthesis and fat synthesis were inhibited. Malonyl-CoA decarboxylase (MCD) regulates the translation of acetyl-CoA from malonyl-CoA, and the expression of its gene, *MLYCD*, was downregulated in the subcutaneous fat of the yaks under ES (*q* < 0.01); thus, acetyl-CoA was decreased. Acetyl-CoA is an initiator of fatty acid oxidation, and the fatty acid oxidation in the AT of the yaks under ES was inhibited.

Energy status regulates cell cycle progression, in part by controlling protein synthesis via mammalian target of rapamycin complex 1 (mTOR) [52,53]. AMPK can inhibit the activity of mTOR [54], and the expression of its gene, *AKT1S1*, was downregulated in the subcutaneous fat of the yaks under ES (*q* < 0.01). The activity of mTOR was inhibited in the subcutaneous fat of the yaks under ES, which can inhibit the activity of downstream serine/threonine-protein kinase ULK1 (ULK1) and eukaryotic translation initiation factor 4E-binding protein 1 (EIF4EBP1), thus regulating cell growth and protein synthesis. The expression of the *ULK1* and *EIF4EBP1* genes was downregulated in the subcutaneous fat of the yaks under ES (*q* < 0.01), and it was inferred that cell growth and protein synthesis in the subcutaneous fat of the yaks under long-term ES were inhibited and energy expenditure was decreased. Moreover, the PI3K-Akt signaling, which is also mainly related to energy metabolism, was also activated [55,56,57]. The expression of the *PIK3R1* gene was upregulated in the subcutaneous fat of the yaks under ES (*q* < 0.05), and the expression of the *AKT1* and *AKT2* genes was downregulated (*q* < 0.01).

## 5. Conclusions

Overall, under long-term energy stress during the cold season, the fatty acid, serum and transcriptome profiles of yaks were completely different from those of the normal yaks. In addition to that, there was a higher level of fatty acid oxidation in the muscles and organs. PUFA synthesis was greater in the subcutaneous fat of yaks. The fat derived from SFAs in the subcutaneous fat of yaks was consumed preferentially in the process of handling ES. Compared with those in yaks under normal physiological conditions, fat catabolism, fat anabolism and fatty acid oxidation in the subcutaneous fat were all lesser in yaks under ES, whereas the transfer of the metabolites of fat catabolism was promoted. Fat cells secreted more of the adipocytokine LEP, and ADP/ATP was greater in the subcutaneous fat of yaks, and then AMPK was activated. Fat metabolism in yaks was inhibited by regulating downstream genes of the AMPK signaling pathway. In addition, the energy consumption for cell growth and protein synthesis in fat cells was inhibited by PI3K-Akt and mTOR signaling, and the muscles and organs in yaks could obtain more energy from the limited GLU and fat. Therefore, the fat utilization efficiency was improved, and yaks could survive long-term ES during the cold season.

## Figures and Tables

**Table 1:** Names, primer sequences and product sizes of the candidate genes.

Gene Symbol	Forward Primer (5′ -> 3′)	Reverse Primer (5′ -> 3′)	Product Size (bp)
*SERPINE1*	CGTCCAGAGAGAGCCACA	ATCCGCATCCTGAATTTCG	118
*FAU*	CTACGCACTCTCGAGGTGA	AGGAGCAGGACTTGATCT	101
*CEBPA*	GCCCGGCAACTCTAGTATTA	TGACAAGGCACATATTTGCT	102
*LIPE*	CTTCTTCGAGGGTGATGAG	CGGGTGTGAACTGGAAAC	107
*ACSF2*	GGCCATCAGCAGAGAAAGA	CACGCATGGTTGAGATGTC	107
*BCKDHA*	ACTTCGTCACCATCTCCT	ACAGATGACCACCCTGTT	101
*GSTM3*	CTCACCTTTGTGGATTTCCTC	ACATGAAAGCCTTCAGATTCG	100
*TMEM165*	AGATGAGTCCAGATGAAGGT	TCAACATCTCCTGGTCCATT	112
*β-actin*	GGATGCAGAAAGAGATCACT	TCTGCTGGAAGGTGGACA	187

**Table 2:** Fatty acid composition (g/100 g identified fatty acid) in the subcutaneous fat of the yaks in the energy stress (ES) group and control (CO) group.

Fatty Acid	Energy Stress (*n* = 6)	Control (*n* = 6)
C4:0	0.17 ± 0.04 ^a^	0.32 ± 0.15 ^b^
C6:0	0.03 ± 0.01	0.05 ± 0.02
C8:0	0.05 ± 0.03 ^A^	0.01 ± 0.00 ^B^
C10:0	0.04 ± 0.01	0.04 ± 0.01
C11:0	0.01 ± 0.00	0.01 ± 0.00
C12:0	0.03 ± 0.00	0.03 ± 0.00
C13:0	0.06 ± 0.03	0.13 ± 0.08
C14:0	0.62 ± 0.13	0.57 ± 0.11
C14:1	0.05 ± 0.01	0.04 ± 0.01
C15:0	0.82 ± 0.03 ^A^	0.33 ± 0.04 ^B^
C15:1	0.41 ± 0.06 ^A^	0.12 ± 0.03 ^B^
C16:0	10.9 ± 0.68 ^A^	14.5 ± 2.23 ^B^
C16:1	1.84 ± 0.12 ^A^	1.43 ± 0.16 ^B^
C17:0	1.97 ± 0.13 ^A^	0.64 ± 0.18 ^B^
C17:1	1.03 ± 0.10 ^A^	0.23 ± 0.13 ^B^
C18:0	22.0 ± 0.87 ^A^	34.8 ± 2.04 ^B^
cis-C18:1	12.9 ± 0.95	12.7 ± 1.19
cis-C18:2	20.0 ± 1.74	19.4 ± 2.43
C20:0	0.15 ± 0.02	0.08 ± 0.03
C18:3n6	1.00 ± 0.17 ^A^	0.22 ± 0.13 ^B^
C20:1n9	0.14 ± 0.04 ^A^	0.39 ± 0.11 ^B^
C18:3n3	2.16 ± 0.45 ^A^	0.84 ± 0.20 ^B^
C21:0	0.43 ± 0.09 ^A^	0.60 ± 0.13 ^B^
C20:2	0.56 ± 0.32 ^a^	0.10 ± 0.04 ^b^
C22:0	0.17 ± 0.11	0.15 ± 0.08
C20:3n3	0.02 ± 0.00 ^A^	0.01 ± 0.00 ^B^
C20:4n6	11.1 ± 0.56 ^A^	4.55 ± 0.38 ^B^
C23:0	0.78 ± 0.28 ^A^	0.19 ± 0.06 ^B^
C24:0	4.45 ± 0.36	4.19 ± 0.43
C20:5n3	3.24 ± 0.64 ^A^	0.84 ± 0.30 ^B^
C24:1	2.60 ± 0.42	2.54 ± 0.73
C22:6n3	0.16 ± 0.07 ^A^	0.02 ± 0.01 ^B^
SFA	42.7 ± 1.46 ^A^	56.6 ± 0.73 ^B^
MUFA	19.0 ± 1.21	17.5 ± 1.83
PUFA	38.3 ± 1.33 ^A^	26.0 ± 2.43 ^B^
UFA	57.3 ± 1.46 ^A^	43.4 ± 0.73 ^B^
SFA/UFA	0.75 ± 0.05 ^A^	1.30 ± 0.04 ^B^
MUFA/PUFA	0.50 ± 0.04 ^a^	0.68 ± 0.13 ^b^
PUFA/SFA	0.90 ± 0.05 ^A^	0.46 ± 0.05 ^B^

Note: Values in the same row with different lowercase superscripts show *p* < 0.05, different capital superscripts show *p* < 0.01.

**Table 3:** The concentrations of metabolites and enzymes in the serum of the yaks in the energy stress (ES) group and control (CO) group.

Item	Energy Stress (*n* = 6)	Control (*n* = 6)
GLU (mmol/L)	3.84 ± 0.12 ^A^	4.95 ± 0.10 ^B^
GC (ng/mL)	89.2 ± 1.50 ^A^	50.3 ± 1.30 ^B^
INS (ng/mL)	9.53 ± 0.52 ^A^	12.0 ± 0.30 ^B^
NEFA (nmol/L)	174 ± 3.20 ^a^	180 ± 3.54 ^b^
TG (mmol/L)	0.19 ± 0.02 ^A^	0.25 ± 0.02 ^B^
HDL (mmol/L)	1.67 ± 0.12	1.77 ± 0.06
LDL (mmol/L)	0.60 ± 0.03 ^a^	0.65 ± 0.03 ^b^
CH (mmol/L)	2.59 ± 0.10	2.70 ± 0.06
OA (mg/100 mL)	13.6 ± 0.93 ^A^	11.2 ± 0.36 ^B^
ACAC (mg/100 mL)	31.8 ± 3.69	34.3 ± 2.47
BHBA (mg/100 mL)	4.26 ± 0.05 ^A^	2.15 ± 0.11 ^B^
HSL (ng/mL)	12.2 ± 1.38 ^a^	9.75 ± 0.18 ^b^
ATGL (ng/mL)	66.9 ± 1.28 ^A^	52.6 ± 1.38 ^B^
ALB(g/L)	26.5 ± 1.02 ^A^	31.2 ± 1.77 ^B^

Note: Values in the same row with different lowercase superscripts show *p* < 0.05, different capital superscripts show *p* < 0.01.

**Table 4:** The kyoto encyclopedia of genes and genomes (KEGG) pathways related to fat metabolism in the subcutaneous fat of yaks under long-term ES.

ID	Term	*p*	Score	ListHits
bom03010	Ribosome	1.23 × 10^−21^	2.50	99
bom00190	Oxidative phosphorylation	9.98 × 10^−16^	2.68	63
bom04714	Thermogenesis	1.45 × 10^−13^	2.14	88
bom04146	Peroxisome	2.35 × 10^−6^	2.17	32
bom04151	PI3K-Akt signaling pathway	3.21 × 10^−5^	1.47	91
bom01040	Biosynthesis of unsaturated fatty acids	3.97 × 10^−4^	2.38	13
bom04512	ECM-receptor interaction	6.69 × 10^−4^	2.77	27
bom04152	AMPK signaling pathway	1.52 × 10^−3^	2.18	25
bom00061	Fatty acid biosynthesis	7.56 × 10^−3^	2.22	7
bom00790	Folate biosynthesis	8.43 × 10^−3^	1.95	10
bom00071	Fatty acid degradation	1.15 × 10^−2^	1.74	13
bom04923	Regulation of lipolysis in adipocytes	1.29 × 10^−2^	1.64	16
bom04920	Adipocytokine signaling pathway	1.46 × 10^−2^	1.57	18
bom00062	Fatty acid elongation	1.47 × 10^−2^	1.87	9
bom00780	Biotin metabolism	1.59 × 10^−2^	3.02	2
bom03320	PPAR signaling pathway	2.44 × 10^−2^	1.46	21
bom04150	mTOR signaling pathway	2.55 × 10^−2^	1.33	35
